# Nappe oscillations on free-overfall structures, data from laboratory experiments

**DOI:** 10.1038/s41597-020-0521-8

**Published:** 2020-06-17

**Authors:** Maurine Lodomez, Blake Tullis, Pierre Archambeau, Vasileios Kitsikoudis, Michel Pirotton, Benjamin Dewals, Sébastien Erpicum

**Affiliations:** 1 Hydraulics in Environmental and Civil Engineering (HECE), University of Liège, Liège, Belgium; 2 Utah Water Research Laboratory (UWRL), Utah State University, Logan, USA

**Keywords:** Civil engineering, Natural hazards

## Abstract

This paper presents a dataset obtained from fifty-two laboratory experiments of nappe oscillations on free overfall structures. Data were collected on two complementary experimental setups, each consisting of a linear weir model. The dataset covers test configurations involving varied geometric parameters (i.e. weir crest shape, weir width, fall height and nappe confinement) and inflow discharges. The following experimental data were produced: assessment of nappe oscillation occurrence and associated frequencies. The later measurements were performed using characterization techniques (image and sound analysis) developed for this research. Reuse of the collected data will support efforts to improve the understanding of the physical processes underpinning nappe oscillation and to validate numerical modelling of the phenomenon.

## Abstract (summary)

**Table:** 

Measurement(s)	nappe oscillation • water flow process
Technology Type(s)	Experimental Model • high-speed motion picture camera • Microphone Device • flow meter
Factor Type(s)	model • weir width • fall height
Sample Characteristic - Environment	laboratory environment

Machine-accessible metadata file describing the reported data: 10.6084/m9.figshare.12338444

## Background & Summary

Free-overfall structures (such as weirs and crest gates) are commonly used as flow control structures for a variety of open channel/free surface flow applications including irrigation, water treatment, and dam safety. The gravity-driven free falling jet on the downstream side of these structures, called the nappe, may display a variety of behaviors and instabilities^1^. In particular, nappe oscillations, also known as nappe vibrations, can occur under relatively low-head discharges and have been observed to occur with a variety of weirs (linear, labyrinth), crest gates and fountains^2–7^.

Frequently identified as undesirable and potentially dangerous, this oscillating-instability phenomenon induces a disturbing noise with unmistakable acoustic energy resulting in low frequency noise that can be heard and felt in the vicinity of the structure^2,3,8^. Nappe oscillations may also lead to structural issues if one of the dominant nappe oscillation frequencies is close to the natural frequency of the structure^9^. In the case of crest gates, the oscillations may cause vibration of the gates and jacks which could lead to technical and operational issues, critical to the sustainability of the structures. Moreover, these oscillations increase the environmental and societal impacts of the hydraulic structure which affect in turn the political acceptance of the work.

A review of the scientific literature^10^ shows that nappe oscillations have been subject to various theoretical^2,11–17^ and experimental^3,6,18–22^ modelling attempts over the last 80 years. It emerges that nappe oscillation is a complex hydraulic process. A number of theories regarding the cause or the origin of the oscillations appearance and development have been put forward. However, no definitive understanding of the phenomenon has been proposed to date. While most experimental research so far focused on vertically falling water sheets generated by a thin slot in a pressurized tube, only few of them explored the nappe oscillation problem associated with free overfall weirs. Such systems, widely used as dam safety structures for flood release, may however experience strong nappe oscillations^6^. Nappe oscillation is a timely topic since many non-linear structures were built in recent years^3^. Such structures due to their improved hydraulic efficiency, operate more frequently under low-head conditions than linear ones and therefore are more often subject to nappe oscillation. An improved understanding of this phenomenon is thus required to better manage its potential occurrences and address the practical consequences.

The present dataset^23^ is the outcome of an experimental research aimed at providing a thorough characterization of nappe oscillation occurring on free overfall structures. A comprehensive test program was conducted (52 tests) on two laboratory setups of varied size scales, shedding light on nappe oscillation occurrence and its characteristics, for varying nappe confinement, weir crest shape, weir width and fall height. Based on distinct acoustic and optical observations of the phenomenon, respectively measured for each test with a microphone and a high speed camera, two original characterization methods have been developed to quantify the nappe oscillations properties. The application of these methods allows determining the occurrence of the phenomenon and identifying of its dominant frequency.

The present dataset complements and comprises findings, including datasets, discussed in three previous research papers by Lodomez et al., focusing on: (i) the characterization methods^24^, (ii) mitigation techniques^25^ and (iii) the size scale effects^26^. Details of the experimental method and scientific analysis of the present dataset were presented in the PhD thesis of Lodomez^10^.

The generated dataset constitutes the first reliable free overfall weir nappe oscillation database for testing theoretical models and validating theories.

## Methods

### Experimental models

**Fig. 1:**
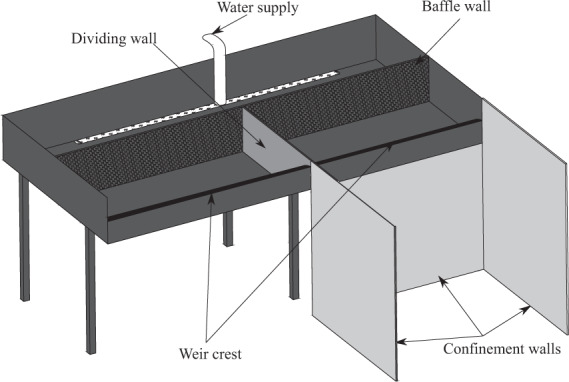
Model 1- Schematic view of the prototype scale model.

**Fig. 2:**
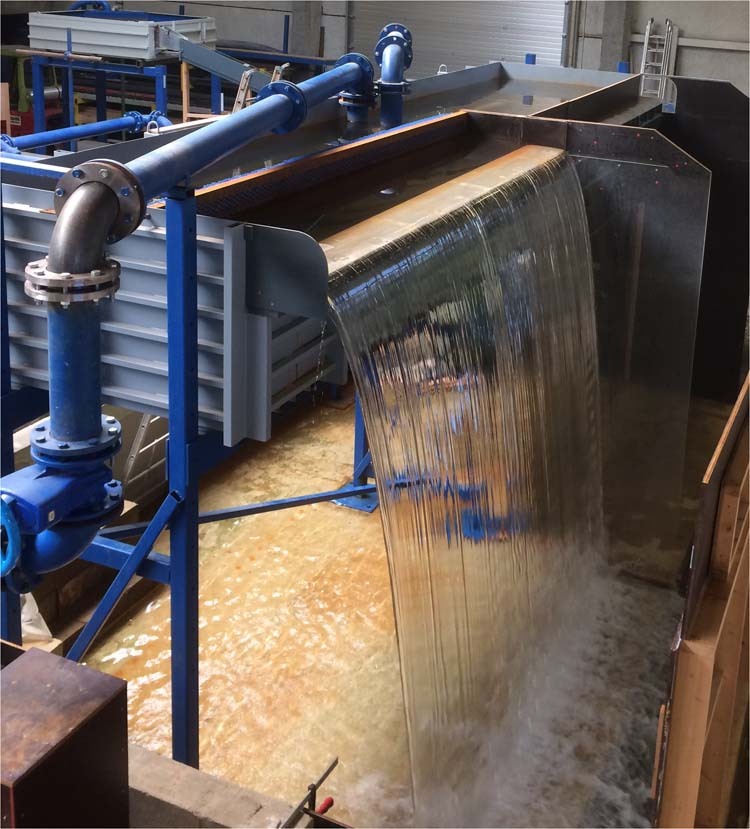
Model 1- View of the prototype scale model with flow over the unconfined weir.

**Fig. 3:**
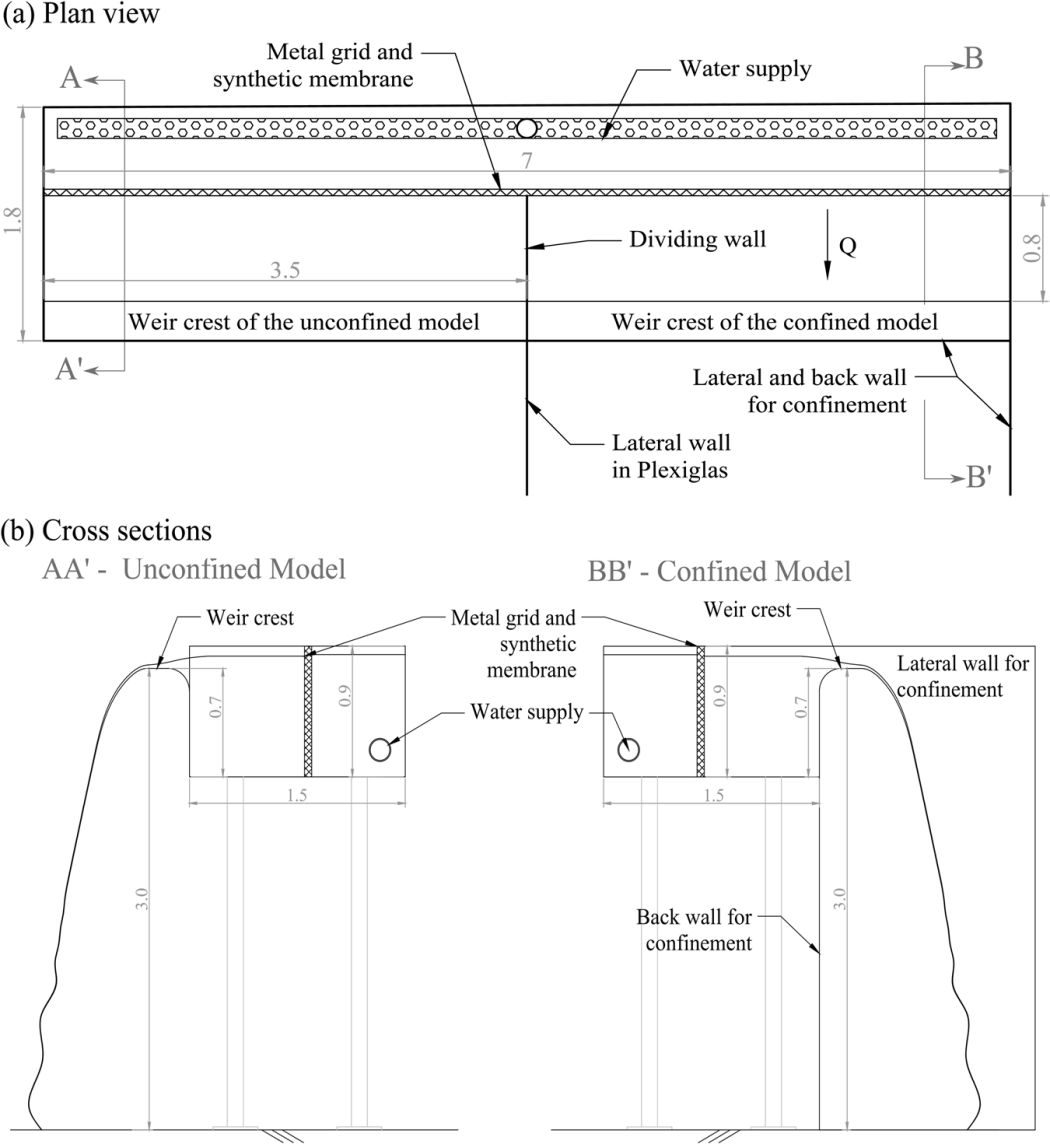
Model 1- Detailed geometry of the prototype scale model. (**a**) Plan view and (**b**) cross sections. Dimensions in m.

Two experimental setups, referred to as Model 1 and Model 2, were specifically designed for this research. Model 1 was a prototype scale model of a linear weir built at the Engineering Hydraulics Laboratory of the University of Liège (HECE - ULiège, Figs. 1–3). It included an elevated headbox that provided flow to two identical weirs installed in parallel. These weirs had a maximum crest length of 3.45 m and a maximum fall height of 3.0 m. The weirs could be isolated so as to only operate one at a time as illustrated in Fig. 2. The headbox was supplied by two pumps delivering up to 0.25 m^3^/s through two perforated pipes parallel to the crest and located on the bottom of the reservoir. Inflows passed through a metal grid and a synthetic membrane to establish uniform approach flows to either weir.

As illustrated in Fig. 3, the weir discharge from the left side of the reservoir was confined downstream with two lateral walls and a back wall, to investigate a confined nappe and a non-vented air cavity behind the nappe. One lateral wall was made of Plexiglas (to enable visual observation) and the others of black multiplex panels. The weir on the right side only included one sidewall and was used for unconfined nappe testing. Therefore, experiments with confined or unconfined air behind the nappe were performed (corresponding respectively to *Confinement* = 1 and *Confinement* = 0 in the dataset^23^).

**Fig. 4:**
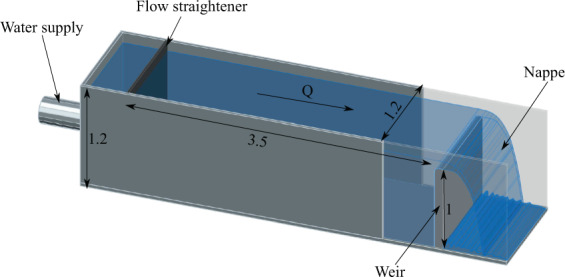
Model 2 – Schematic view of the small scale model. Dimension in m.

**Fig. 5:**
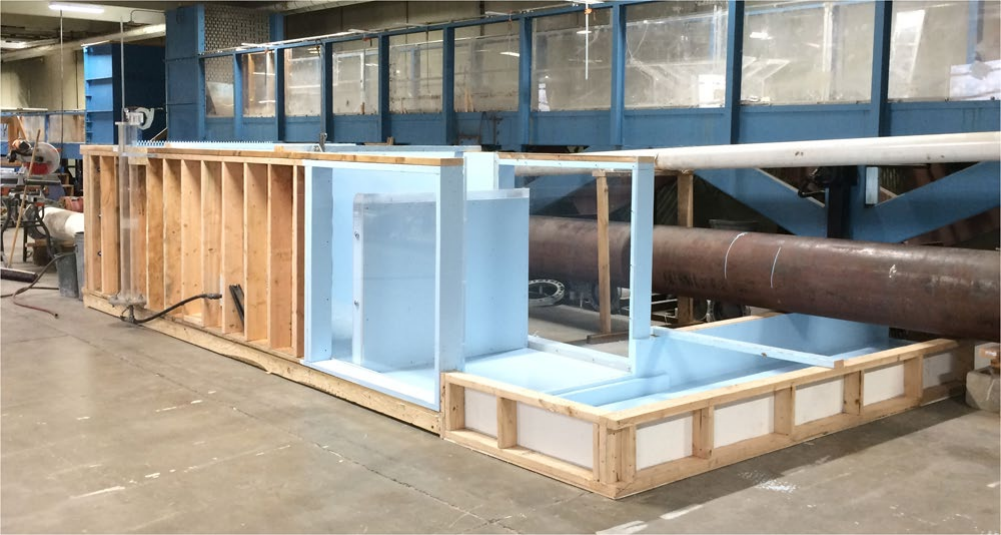
Model 2- View of the small scale model.

To investigate the potential of scale effect^26^ related to weir size, a smaller Model 2 was built and tested at the Utah Water Research Laboratory (UWRL) of Utah State University (USU). It was a 1.2 m wide linear weir placed in a rectangular flume 4.7 m long, 1.2 m wide and 1.2 m deep (Figs. 4 and 5). The flume was supplied with water up to 0.075 m^3^/s via a 0.3-m diameter pipe which contained a calibrated orifice plate flowmeter. A flow straightener and a baffle wall were placed near the upstream end of the flume to uniformize the velocity distribution and then improve flow conditions. The linear weir, which consisted of a wooden frame with plywood sheeting and an acrylic crest, was installed 3.5 m downstream from the flume inlet. The fall height was 1.0 m and the nappe and downstream flow were confined by two sidewalls (acrylic) and a back wall (wood). All the tests with Model 2 were done with the confined configuration.

**Fig. 6:**
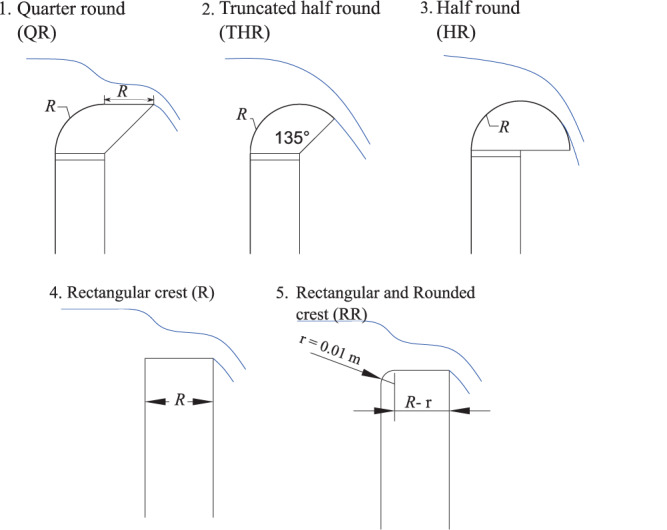
Schematic of the crest shapes.

The five weir crest shapes tested during this study, as illustrated in Fig. 6, were evaluated during this study. In the dataset, the crest shapes are numbered from 1 to 5 as follow: 1. quarter round (QR), 2. truncated half round (THR), 3. half round (HR), 4. rectangular (R) and 5. rectangular and rounded (RR) crest. For Model 1, the crest shapes 1, 2 and 3 were tested with a characteristic dimension *R* equal to 0.15 m. For Model 2, the crest shapes 1, 2, 4 and 5 were tested with a characteristic dimension *R* was equal to 0.05 m.

In addition to the crest profile, the width and fall height were varied. For width modifications, a false wall was placed in the approach channel and a wooden movable downstream apron was used to reduce the fall height. All the configurations tested during this research are reported in the Online-only Table 1.

### Measurement devices

The oscillation of the thin flow nappe cascading downstream from the weir crest and the noise production are respectively the visible and audible characteristics of the nappe oscillations. These characteristics were recorded respectively by the use of a high speed camera and a microphone, and analyzed by a specific method developed in this research. Additionally, hydraulic characteristics of the flow, i.e., discharge and water levels, were measured using a flow meter and point gauges.

#### Microphones

To get data on the noise produced by the oscillations, a free-field microphone, MC212 (01dB-Metravib, Limonest, France) or Behringer ECM 8000 (Behringer GmbH, Willich, Germany) depending on the test, was placed along the centerline of the weir crest, 2.5 m downstream in front of the falling nappe. After testing several positions around the model, it was found that placing the microphone in front of the nappe as specified above makes it possible to obtain, on the one hand, the sound that is least affected by ambient noise, in particular the noise generated by the pumps and, on the other hand, the most direct sound produced by the oscillations. The MC212 and Behringer ECM 8000 microphones have respectively a frequency range of 6 Hz to 20 kHz and 15 Hz to 20 kHz. The recording of audio data was carried out for the MC212 microphone with dBFA from the dB1-Metravib software suite and with SIGVIEW software for the Behringer microphone. Both microphones were calibrated by the Cedia, the research department of acoustic and vibrations of ULiège. For each test, i.e. specific geometric configuration and unit discharge (*q*), the sound measurements were performed for a period of 2 minutes (with MC212 microphone) or 4 minutes (with Behringer ECM 8000 microphone) and repeated at least twice.

#### High speed cameras

**Fig. 7:**
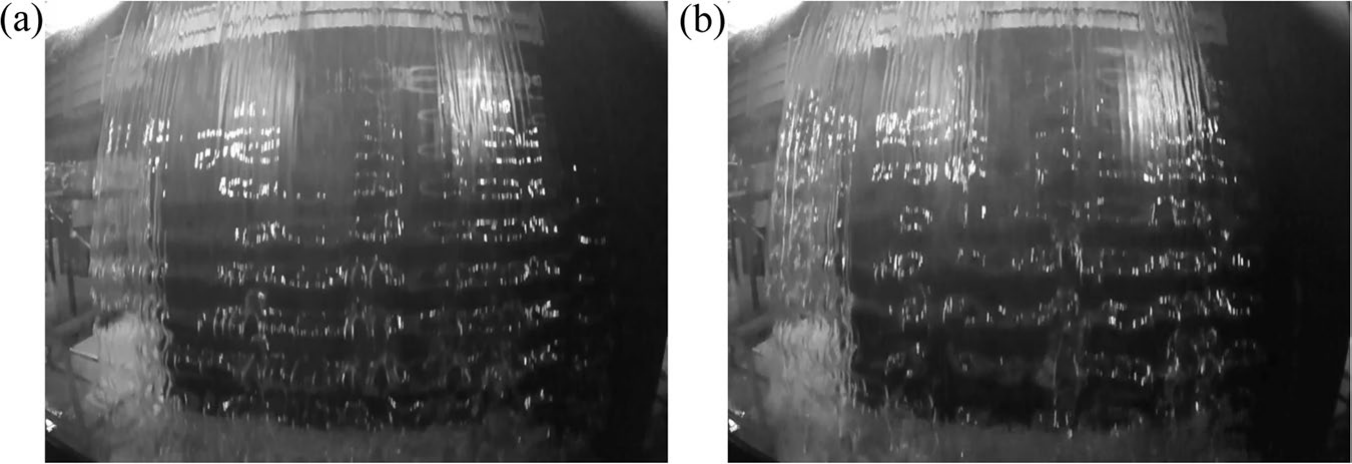
Nappe visualization for the Test n°1 (**a**) *q* = 0.02 m^2^/s and (**b**) *q* = 0.03 m^2^/s (images recorded at 240 frame per second with camera Go-Pro Hero 4).

The second method used to characterize the nappe oscillations is based on image analysis. For this purpose, two high speed cameras, Go-Pro Hero 4 (GoPro, San Mateo, California) and ImagerMX4M Lavision (Lavision, Goettingen, Germany), were available in the Laboratory. As both cameras have an acquisition frequency higher than 200 Hz, they allow capturing the visible oscillations until a maximum oscillating frequency of 100 Hz according to the Nyquist-Shannon sampling theorem^27^. Assuming the frequencies of interest in this study were lower than this limit of 100 Hz, both cameras were used indifferently depending on their availability. Horizontal bands being the visible characteristics of the nappe oscillation phenomenon, the camera was placed along the centerline of the weir crest, 2.5 m downstream in front of the falling nappe. Using an appropriate lighting, this location allowed the capture of the visible horizontal bands on the images of the falling water, as illustrated in Fig. 7.

#### Flowmeters

The discharge provided to the model was the main hydraulic parameter controlled by the experimenter through the opening or closing of valves and adjustment of pump rotation speed. For Model 1, the measurement of this parameter was performed with a flowmeter Endress + Hauser PROMAG 50 W (Endress + Hauser NV/SA BeLux, Bruxelles, Belgium) connected to the main supply pipe, of 300 mm in diameter. The accuracy of the flowmeter Endress + Hauser is +/−0.5% of the full scale (0.4 m^3^/s) according to the calibration sheet of the device, which is +/−2 × 10^−3^ m^3^/s. For Model 2, an orifice plate, connected to the 203.2 mm diameter supply pipe, was used. This measurement tool was calibrated and had a total uncertainty of +/−0.25% of the discharge, which gave for the maximum tested discharge an accuracy of +/−2 × 10^−4^ m^3^/s. In this research, the use of a unit discharge $q=\frac{Q}{W}$ in m^2^/s, ratio of the total discharge *Q* (m^3^/s) to the crest width *W* (m), has been preferred to the total discharge *Q*. The maximum *q* tested was 0.07 m^2^/s for Model 1 and Model 2 which corresponds respectively to 241.5 × 10^−3^ m^3^/s and 84.0 × 10^−3^ m^3^/s.

#### Point gauges

Water levels, in the reservoir and on the crest, were acquired for each discharge and crest shapes 1, 2, 4 and 5. For Model 1, measurements of the upstream water level (*h*_*u*_) were performed in the headbox 65 cm upstream of the weir using a point gauge located about 20 cm from the dividing wall. The water level at the flow detachment (*h*_*c*_, at the crest downstream extremity) was also measured with a point gauge. The measurement accuracy was +/−1 mm. For Model 2, a point gauge was placed 1.5 m upstream of the crest downstream end while a second one was movable from 50 cm upstream to the downstream apex of the crest. Water level measurements for this model were performed with an accuracy of +/−0.6 mm. The upstream water depths reported in the dataset correspond to water depths measured upstream, positively over the top of the crest. For the water level at the nappe detachment point, the point gauge allowed to measure vertically the nappe thickness. In that case, the reference over which the water is measured was the crest level (at the detachment).

### Data processing and characterization of the nappe oscillations

To collect relevant and quantitative data from sound measurements and videos, specific analysis methods were developed^10^. These two methods allowed the definition of the oscillation occurrence and a quantification of the main characteristics provided in the dataset^23^.

#### Sound analysis

**Fig. 8:**
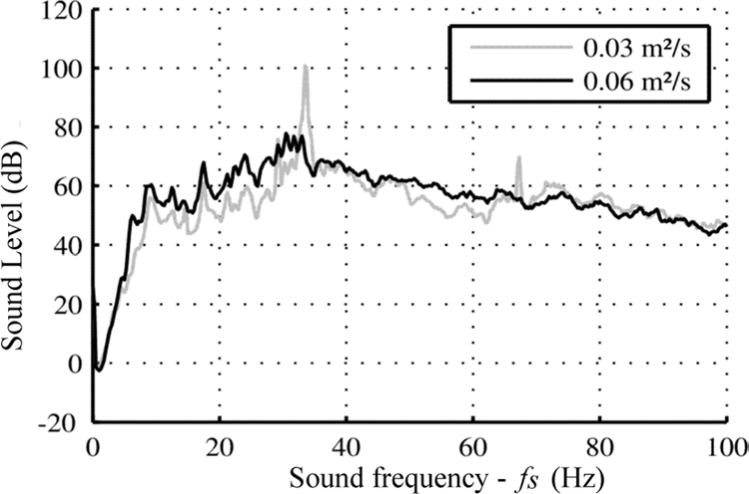
Mean auto-spectrum of audio signal for Test n°1, *q* = 0.03 m^2^/s and *q* = 0.06 m^2^/s.

The first method used to assess the occurrence of the nappe oscillation is based on a sound analysis since the noise production is a noticeable characteristic of nappe oscillations. Based on sound measurements (time evolution of air pressure), the sound analysis was carried out by the software used for the recording, i.e. dBFA (https://www.01db.com/) or SIGVIEW (https://www.sigview.com/). The dBFa software supplied the auto-spectrum of the audio signal by applying a narrow-band analysis. The audio auto-spectrum provides an image of the sound level for each frequency of the noise. This spectral analysis is based on a standard periodogram or Gabor analysis, with the selection of an overlap and a weighting window^28^. The result, named sound level or intensity in the following discussion, is expressed in dB and is equal $2{0\log }_{10}\frac{\left|{G}_{xx}\right|}{2.1{0}^{-5}}$ with the modulus of the complex auto-spectrum. Based on audio recording, this spectral analysis was applied considering an overlap of 50%, a Hanning window and a frequency resolution of 0.25 Hz. The analysis resulted in a narrow-band multi-spectrum with a time step of 125 ms. This result was exported as a text file and analyzed with MatLab software. As developed in Lodomez *et al*.^10,24^, the representation of the narrow-band multi-spectrum in time showed that this acoustic property was stationary whatever the configuration or *q*. This spectrum provided an image of the sound level for each frequency of the noise generated by the nappe cascading downstream from the weir. The SIGVIEW software provided directly the mean auto-spectrum with a frequency resolution of 0.01 Hz with the application of the same filtering (Hanning window). Therefore, the sound signal processing generated a mean auto-spectrum, called simply spectrum. From this result, two quantitative parameters were extracted to characterize the oscillations: the maximum sound level (*I*) and its associated frequency (*f*_*s*_) as illustrated in Fig. 8 for Test n°1 and two values of *q*. Finally, this spectrum allowed us to determine the occurrence or non-occurrence of the nappe oscillation, which is characterized by the presence of a local peak in the spectrum. Indeed, Lodomez *et al*.^10,24^ showed that the spectra extracted from sound measurements were typically of two types, as illustrated in Fig. 8. The figure shows, for *q* = 0.03 m^2^/s, a spectrum in which a clearly visible peak in sound level appears for a specific frequency while for *q* = 0.06 m^2^/s, there is no obvious dominant peak. For *q* = 0.03 m^2^/s, the maximum quantifies the magnitude of the phenomenon while the frequency of the oscillations was directly given by the associated frequency of the peak in the audio spectrum. For *q* = 0.06 m^2^/s, nappe oscillations were weak or non-existent as there was no local maximum in the spectrum.

In the present dataset^23^, for each test configuration reported in Online-only Table 1, each measurement is reported in the same order as it has been performed. The data reported is a vector of unit discharges (*q*_*s*_) to which are associated the vectors of the maximum sound level extracted from spectrum (*I*), the occurrence assessment of nappe oscillations [*O*_*s* = _1 or 0 respectively in case of nappe oscillations or not (this assessment has been performed manually by M.L.^10^ based on the analysis of the spectrum for each unit discharge)] and finally the frequency in case of nappe oscillation occurrence (*f*_*s*_). For each test, the data takes thus the form of four vectors.

#### Image analysis

**Fig. 9:**
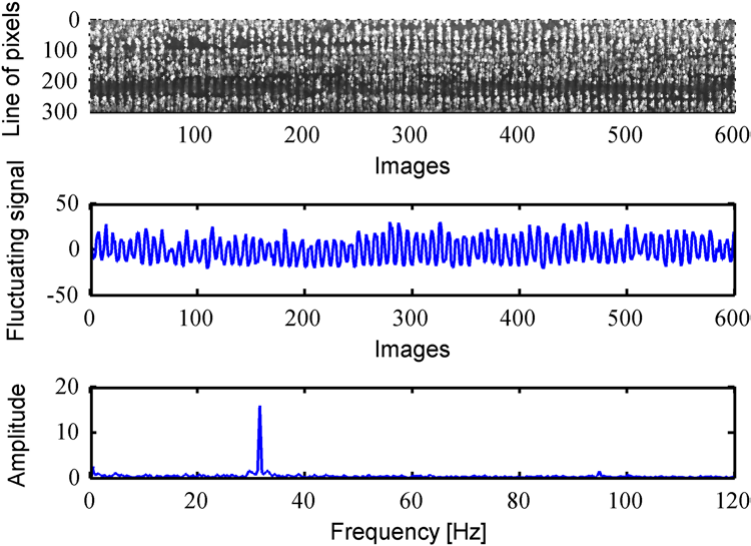
Steps of image analysis on 600 images for the Test n°26 and *q* = 0.045 m^2^/s.

As shown in Fig. 7, the viewing with a high speed camera of an oscillating nappe allows the detection of horizontal bands. They are the visible characteristics of the nappe oscillation as referred in literature^2,3^. In addition, the analysis of successive images shows the propagation of the oscillations along the nappe according to the flow trajectory^24^. The image analysis procedure has been developed to quantify the frequency related to horizontal bands displacement along the nappe. Assuming that the horizontal bands are due to the lighting on the undulating surface, the frequency of the bands apparition at a given location along the nappe trajectory was determined by the time evolution of data carried by a set of pixels on a sequence of raw images^10^. For a fixed image frame, a chosen line of pixels carries information which varies in time. The numerical value associated to each pixel depends on whether the pixels are on the illuminated zone of the oscillation or not, which is a function of the curvature of the nappe undulation (concave or convex). This numerical value, for images in grayscale and encoded in 8 bits, is between 0 (white) and 255 (black). Considering the average value on a line of pixels, the oscillation frequency is calculated by the Fast Fourier Transform (FFT) of its time evolution. In order to illustrate this methodology, an analysis on 600 images (recorded at 240 Hz, i.e. 2.5 s duration movie) is shown in Fig. 9 for Test n°26 and *q* = 0.045 m^2^/s. This figure illustrates (1) the time evolution of a chosen line of 300 pixels, (2) the fluctuation of the average pixel value on this line and (3) the FFT of this fluctuating signal. This method therefore links the detection of an oscillation on an instantaneous picture to an oscillating signal (in time) as illustrated by the time evolution of the chosen line, and finally to an oscillation frequency. The resolution of this frequency calculation is given by the ratio between the acquisition frequency and the number of snapshots. The acquisition frequency of the camera was either 240 Hz for the Go-Pro Hero4 or 300 Hz for the ImagerMX4M. Image analysis was conducted with the number of snapshots needed to get a resolution at least identical to that of sound analysis, i.e. 0.25 Hz.

The image analysis was performed, for each test configuration, for *q* between 0.01 and 0.06 or 0.07 m^2^/s with a step of 0.01 m^2^/s. A minimum of six lines of 300 pixels were considered per snapshot ant at least 5 sets of images were analyzed for each test configuration and *q*. For a set of images, the application of the method showed that the frequencies extracted from the different lines did not vary by more than the accuracy of the method, i.e. 0.25 Hz.

Based on the analysis of all *q*, the dataset^23^ reports first the occurrence of the nappe oscillation by means of a variable *O*_*i*_ (*O*_*i* = _1 if nappe oscillations have been detected for at least one tested unit discharge, *O*_*i* = _0 no nappe oscillation was detected for this test configuration). Then, in case of nappe oscillation occurrence, two vectors are provided: (i) the unit discharges in ascending order and (ii) the associated frequencies of the horizontal bands (*f*_*i*_).

## Data records

### Data structure

The present dataset^23^ is structured as presented in Online-only Table 1. All collected data are stored in a single file under HDF5 format (Hierarchical Data Format 5). HDF5 libraries exist for different programming languages *e.g*. Python, Matlab, Fortran, C and R). More details can be found on the HDF5 website (https://support.hdfgroup.org/HDF5/).

Data stored in the HDF5 file are structured following a directory, subdirectory, and file tree structure, corresponding to groups, subgroups and datasets, respectively (https://support.hdfgroup.org/HDF5/). The data are organized under 52 groups corresponding to the 52 tested configurations. For each group, the data consist of four subgroups namely: (i) experimental configuration, (ii) sound analysis, (iii) image analysis and (iv) water depth. The subgroup “experimental configuration” summarizes the model characteristics and dimensions. The subgroup “sound analysis” reports the results of the sound analysis by means of four vectors, as detailed above: for each tested *q*_*s*_, the maximum sound level (*I*) of the spectrum is provided as well as the nappe oscillation occurrence assessment (*O*_*s*_) and the sound frequency in case of occurrence. The subgroup “image analysis” reports the results of the image analysis by means of one variable *O*_*i*_ assessing the occurrence of nappe oscillation for the tested configuration and, in case of nappe oscillation occurrence, two vectors *q*_*i*_ and *f*_*i*_ reporting the unit discharges impacted by nappe oscillations and the associated frequencies extracted from image analysis.

**Fig. 10:**
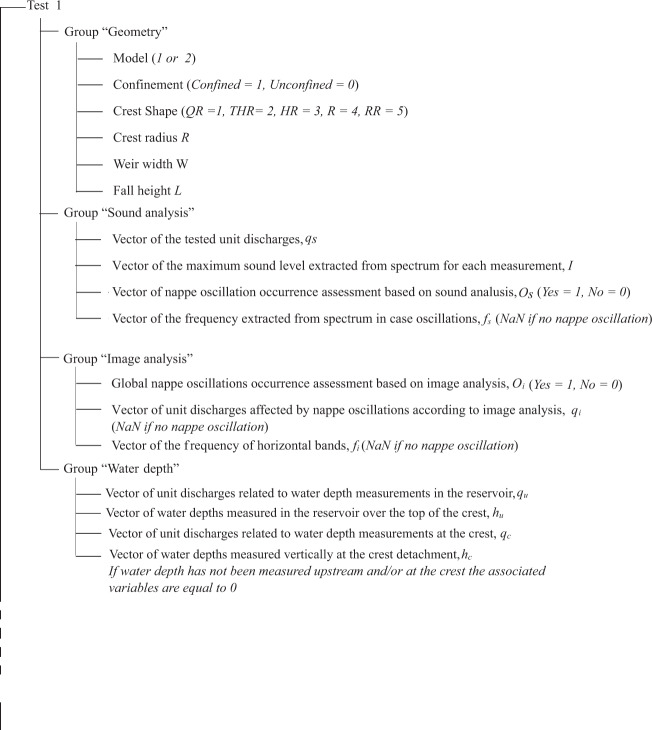
Example of data structure, corresponding to Test 1.

Finally, the subgroup “water depth” reports for the test configurations n°1, 2, 45, 48 and 52, respectively the unit discharge *q* and the water depth *h* measured upstream (subscript *u*) and at the crest detachment (subscript *c*). An example of the data structure is illustrated for Test 1 in Fig. 10. Complementary raw data are also provided^29^ for all 52 cases in the form of video and audio recordings. Specifically, for each case there are mp4 video files for unit discharges ranging from 0.01 m^2^/s to 0.06 m^2^/s with increments of 0.01 m^2^/s, while the respective audio data are either wav audio files or txt files associated to the narrow-band multi-spectrum with a time step of 125 ms that was generated by the software for each measurement. In cases where measurements were conducted for a more limited range of unit discharges, only the available raw data are provided.

### Notations and units

Datasets in the HDF5 are labelled following the physical variable name. Table 1 lists names of variables and associated units.

**Table 1:** Dataset notations and units.

	Label in the dataset	Variables		Units
Experimental configuration	Model	1 (Model 1) or 2 (Model 2) 1 (confined) or 0 (unconfined) 1 (QR), 2 (THR), 3 (HR), 4 (R), 5(RR)		(—)
	Confinement			(—)
	Crest			(—)
	R	*R*	Characteristic crest dimension (Fig. 6)	(m)
	W	*W*	Weir width	(m)
	L	*L*	Fall height	(m)
Sound analysis	q_s	*q*_*s*_	Vector of the tested unit discharges	(m^2^/s)
	I	*I*	Vector of maximum sound levels	(dB)
	O_s	*O*_*s*_	Vector of occurrence *(1 = nappe oscillation, 0 = no nappe oscillation)*	(—)
	f_s	*f*_*s*_	Vector of sound frequency (NaN if no nappe oscillation)	(1/s)
Image analysis	O_i	*O*_*i*_	Occurrence of nappe oscillation for at least one tested *q* based on image analysis *(1 = nappe oscillation, 0 = no nappe oscillation)*	(—)
	q_i	*q*_*i*_	Vector of unit discharges *(NaN if no nappe oscillation)*	(m^2^/s)
	f_i	*f*_*i*_	Vector of frequency extracted from image analysis *(NaN if no nappe oscillation)*	(1/s)
Water depth	q_u	*q*_*u*_	Vector of unit discharges associated to *h*_*u*_	(m^2^/s)
	h_u	*h*_*u*_	Vector of upstream water depths	(m)
	q_c	*q*_*c*_	Vector of unit discharges associated to *h*_*c*_	(m^2^/s)
	h_c	*h*_*c*_	Vector of water depths at the crest detachment	(m)
			*Vectors will be a variable equal to 0 if no measurement has been performed*	

## Technical validation

The validation of the dataset is divided in three parts. The first two parts cover the validation of the measurements and characterization techniques of the nappe oscillation based on sound and image analyses. The third part focuses on the comparison of the characterization techniques.

### Sound measurement and sound analysis

The sound data reported in the dataset^23^ have been collected and processed by means of two different microphones and software. Lodomez^10^ showed that for identical test configurations and *q*, both measurements tools provided similar results in term of spectra. However, the intensity of the spectrum varies due to the use of different microphones. More generally, sound intensities extracted from spectra are not comparable if the sound signals were recorded with different microphones and/or in different acoustic environments, i.e. different laboratories for instance. Therefore, to compare sound intensities recorded with different microphones and processed with different software, the intensities measured for a defined configuration (i.e., for fixed confinement and geometric parameters) should be normalized, for instance using the maximum intensity measured for the tested configuration^10^.

The oscillations were not the only process able to create a local peak in the audio spectrum. Acoustic recordings also included background noise, in particular the pump one, with other main frequencies, in particular the frequency associated to the pump rotation. To overcome this problem, special care has been paid in every test to adapt the pump frequency so as not to match the frequency of other local peaks in the spectrum. In addition, when repeating the tests, different pump frequencies were used to isolate unequivocally the sound signal from the oscillations. Therefore, all the intensities and associated frequencies of spectra presented in this study are unequivocally related to the nappe oscillation.

Finally, the analysis presented above to characterize the nappe oscillation is based on the assumption that the oscillations noise results from the action of the nappe on the surrounding air and not from the impact on the apron. This assumption has been verified by Lodomez^10^ with an analysis of the sound spectrum for various impact conditions.

### Nappe oscillation visualization and image analysis

First of all, although two cameras were available, nappe oscillations were almost always captured with the GoPro for practical reasons. However, the visualization of the horizontal bands may be performed with any other camera provided that the acquisition frequency is adequate (usually higher than 100 Hz). The horizontal bands are indeed largely documented in the literature^2,18,22^.

Second, the frequency of these horizontal bands reported in the dataset is validated by the duplications of image analysis. Indeed, for each test and *q*, a minimum of 6 lines of 300 pixels were considered per snapshot and at least 5 sets of images were analyzed. Therefore, the image analysis led to a minimum of 30 calculated signals per tested unit discharge. The horizontal lines of pixels taken into consideration for the image analysis were distributed evenly along the frame. The application of the methodology for various configurations showed that a better detection of the bands was obtained for the lines positioned near the crest (within a distance of 1 m), due to the fact that the nappe is more uniform close to the detachment location. However, data collected for lines positioned far downstream from the crest provided identical results in term of frequency, only the amplitude of the peak in the FFT was affected by the position of the line. The number of lines was set at 6 to get robust data and may be increased depending of the fall height of the nappe. Finally, the choice of a minimum of 300 pixels per line has been fixed to correspond to the entire width of the narrowest nappe. The position of the camera being fixed; the number of pixels was increased according to the width of the nappe so that the line corresponds to the nappe width or the full image width.

### Comparison of the characterization techniques and parameters

The sound analysis and the image analysis being two independent procedures, the results extracted from these methods were compared in details by Lodomez^10^. First of all, for all test configurations, both analysis detected oscillations in a similar flow range. Second, both methodologies provided globally the same oscillations frequency. Nevertheless, it happens that in some case image analysis did not provide all the distinct frequencies found by sound analysis or vice versa.

Lodomez^10^ presented a detailed comparison of the oscillation frequencies collected by sound and image analyses for Test n°1, 2 and 26. It was shown that sound and image analyses provide, for a fixed *q*, oscillation frequencies equal within a 2.5 Hz variation range.

A last validation of the collected data is provided by literature as, using similar geometric parameters, Sumi & Nakajima^20^ and Anderson & Tullis^22^ observed nappe oscillation frequencies in the same range of *q*.

Finally, the two characterization techniques developed in this research proved their robustness by an application in the field^7^.

### Data usage caution

The water depths measurements were performed for Test n°1, 2, 45, 48, 51 and 52. They correspond to the crest shapes 1 and 2 with two distinct crest dimensions (Tests n° 1, 2, 45, 48), as well as crest shape 4 (Test n° 51) and 5 (Test n°52). The resulting relations between the (unit) discharge and the upstream head may be extended, for a similar crest profile and identical *R*, to all configurations which differ in terms of width, fall height and confinement, since these parameters have no influence on the discharge capacity of the weir.

The water and air temperatures were not systematically measured during the tests. However, the air temperature for tests performed with Model 1 as well as the water temperature should vary only slightly over the measurement durations since the ULiege laboratory is heated. The temperature varies between 19 and 25 °C over the year and the water circulates through a closed loop at a mean temperature around 15 °C (underground reservoir). In contrast, for Model 2, the temperature of the water coming from the river next to the UWRL was measured and was equal to 3.5 ± 0.5 °C during all tests. The air temperature has not been measured but is estimated to be 17 °C ± 2 °C.

## Usage notes

This dataset is intended to bring insights into nappe oscillation characteristics in particular those affecting free overfall structures. A large amount of nappe characteristics, in term of discharge range of occurrence, noise production and oscillations frequency, as well as associated geometric and hydraulic parameters, were collected. This dataset is expected to prove particularly valuable since only scarce data were available in literature and only few of them provide sufficient quantitative information in terms of geometry and hydraulic features as well as discharge range of occurrence and associated frequencies.

## Data Availability

The characterization techniques were developed on Matlab 2017b. Futher details on these methods are given by Lodomez^10^.

## Online-only Table

**Online-only Table 1:** Overall test program, The values bracketed are the numerical values associated to model, confinement or crest shape type as reported in the dataset.

Test n°	Model	Confinement	Crest shape	R	W	L
1	Uliège model (1)	Confined (1)	QR (1)	0.15	3.45	3
2			THR (2)	0.15	3.45	3
3						2.5
4						2
5						1.5
6						1
7					2.5	3
8						2.5
9						2
10						1.5
11					2	3
12						2.5
13						2
14						1.5
15					1.5	3
16						2.5
17						2
18						1.5
19					1	3
20						2.5
21						2
22						1.5
23			HR (3)	0.15	3.45	3
24						1
25						0.5
26		Unconfined (0)	QR (1)	0.15	3.45	3
27						2.5
28						2
29						1.5
30						1
31						0.5
32					2.45	3
33						2.5
34						2
35						1
36						0.5
37					2	2
38						1.5
39						1
40						0.5
41					1.5	3
42						2.5
43						2
44					1	3
45	UWRL model (2)	Confined (1)	QR(2)	0.05	1.2	1
46						0.75
47						0.5
48			THR (2)	0.05		1
49						0.75
50						0.5
51			R (4)	0		1
52			RR (5)	0.01		1
